# Deep Convolutional Neural Network-Based Lymph Node Metastasis Prediction for Colon Cancer Using Histopathological Images

**DOI:** 10.3389/fonc.2020.619803

**Published:** 2021-01-13

**Authors:** Min Seob Kwak, Hun Hee Lee, Jae Min Yang, Jae Myung Cha, Jung Won Jeon, Jin Young Yoon, Ha Il Kim

**Affiliations:** ^1^ Department of Internal Medicine, Kyung Hee University Hospital at Gangdong, Kyung Hee University College of Medicine, Seoul, South Korea; ^2^ Department of Computer Science and Engineering, Konkuk University, Seoul, South Korea

**Keywords:** prognostic score, deep learning–artificial neural network, colorectal cancer, metastasis, histology

## Abstract

**Background:**

Human evaluation of pathological slides cannot accurately predict lymph node metastasis (LNM), although accurate prediction is essential to determine treatment and follow-up strategies for colon cancer. We aimed to develop accurate histopathological features for LNM in colon cancer.

**Methods:**

We developed a deep convolutional neural network model to distinguish the cancer tissue component of colon cancer using data from the tissue bank of the National Center for Tumor Diseases and the pathology archive at the University Medical Center Mannheim, Germany. This model was applied to whole-slide pathological images of colon cancer patients from The Cancer Genome Atlas (TCGA). The predictive value of the peri-tumoral stroma (PTS) score for LNM was assessed.

**Results:**

A total of 164 patients with stages I, II, and III colon cancer from TCGA were analyzed. The mean PTS score was 0.380 (± SD = 0.285), and significantly higher PTS scores were observed in patients in the LNM-positive group than those in the LNM-negative group (*P* < 0.001). In the univariate analyses, the PTS scores for the LNM-positive group were significantly higher than those for the LNM-negative group (*P* < 0.001). Further, the PTS scores in lymphatic invasion and any one of perineural, lymphatic, or venous invasion were significantly increased in the LNM-positive group (*P* < 0.001 and *P* < 0.001).

**Conclusion:**

We established the PTS score, a simplified reproducible parameter, for predicting LNM in colon cancer using computer-based analysis that could be used to guide treatment decisions. These findings warrant further confirmation through large-scale prospective clinical trials.

## Introduction

Colon cancer is a major cause of morbidity and mortality worldwide, and its occurrence is expected to increase significantly over the next few years (1, 2). In recent years, the number of dysplastic and colon cancer cases has increased, resulting in increased awareness and the introduction of screening and surveillance programs for colon cancer (3, 4). The presence of lymph node metastasis (LNM) is a crucial prognostic factor to determine whether patients with early-stage colon cancer should undergo additional surgery after local endoscopic treatment and whether adjuvant chemotherapy is necessary after surgical resection for those in the advanced stages (5–7).

Currently, clinicians make important treatment decisions through nodal status evaluation based only on limited radiological examinations, such as ultrasound and computed tomography, and on manual evaluations of a few histological features *via* light microscopy. However, qualitative evaluation of pathological features exclusively (such as histologic type, depth of tumor invasion, and tumor grades) is insufficient for predicting the presence of LNM in patients with colon cancer; inconsistent determinations among experienced pathologists have been noted even with the best-characterized histopathological features. Furthermore, micro-metastasis (8, 9), the presence of minimal cancer cells in regional lymph nodes that pathological examination cannot detect, is observed through immunohistochemistry and molecular genetic evaluation in up to 50% of patients with node-negative colon cancer even after radical surgery, aside from local endoscopic treatment being unable to provide an accurate status of regional lymph nodes (10–12).

Recently, computer-aided image analysis in histopathology evaluations has been shown to offer efficient, accurate, and consistent quantitative feature extraction and also provides decision-making support to ensure diagnostic consistency (13, 14).

Therefore, the purpose of this study is to identify a pathological parameter for reliable and accurate assessment of LNM using a deep convolution neural network (CNN) model that can better stratify patients with colon cancer.

## Materials and Methods

### Histopathology Image Resource for Predictive Parameter

All available digital whole-slide stained high-resolution histopathological images of colorectal cancer (CRC) were obtained from the Genomic Data Commons Data Portal of the National Cancer Institute (https://portal.gdc.cancer.gov/). Pathology slides for the presence and extent of tumors and various tissue components were manually reviewed by a board-certified pathologist (K.Y.W.). The number of pathology image slides publicly available from The Cancer Genome Atlas (TCGA) cohort varied, ranging from one to eight slides (a majority of patients had only one representative slide of the tumor specimen). At model inference and evaluation, only one representative slide for each patient was used in the analysis. Patients with rectal cancer were excluded because rectal cancer differs from colon cancer in its outcome patterns, such as local relapse or metastasis in the disease course after curative surgery (15). Slides with tissue folds, torn tissues, inadequately stained tissues, or other artifacts as well as slides without any tumor tissue were excluded. Based on the American Joint Committee on Cancer (AJCC) staging system, the patients with colon cancer were divided into LNM-positive (stage III) and LNM-negative (stage I and stage II) groups, according to the pathological presence or absence of LNM (16), respectively. Extramural tumor deposits (EMTDs), including lymphatic invasion (LI), venous invasion (VI), perineural invasion (PI), and any of the aforementioned features (AnyI), were also evaluated (17). This study was reviewed and approved by the Institutional Review Board of the Kyung Hee University Hospital at Gangdong (KHNMC IRB 2020-09-025). The need for informed consent was waived because all data used in this study were de-identified.

### Training and Testing of Neural Networks

For the training of our CNN model, we used a training image set comprising 100,000 image patches (224 × 224 pixels and 0.5 μm/pixel), with an approximately equal number of images for the following seven tissue classes: normal colon mucosa, stroma, lymphocytes, mucus, adipose tissue, smooth muscle, and colon cancer epithelium; this image set is publicly available at http://dx.doi.org/10.5281/zenodo.1214456. We conducted the image segmentation using the multi-threshold technique (18). Our network architecture for the auto-segmentation of tumor-microenvironment-related features in colon cancer histology is based on the U-Net architecture (19) because this architecture was initially proposed to improve the performance of fine segmentation and localization, particularly for biomedical images. For images with heights and widths less than the target size, we padded all image patches with “reflect padding” to obtain a size of 512 × 512; the padded voxels were acquired by mirroring the existing images. All images were normalized using the Macenko method (20) and were preprocessed prior to thresholding *via* histogram normalization, in order to standardize the intensities of each RGB channel in the range of 0 to 255 (21). The threshold values were empirically selected, and visual validation was conducted by an experienced pathologist. Among the generated image patches, 80% were used to train our model with forward and backward propagation. To build high-performance network architectures, we divided the remaining data into a 10% validation set and 10% testing set. We used Adam to minimize the cross-entropy loss during stochastic optimization and the adaptive momentum algorithm for smooth convergence (22). Training was terminated when the mean Dice similarity coefficient (DSC) for the validation dataset did not increase by at least 0.1% after 10 additional epochs from its epoch with the best performance. The best model was generated in the epoch with the highest mean DSC. Training was implemented with the Keras library on a parallel computing architecture, using an Intel Core i9-7960 CPU (2.8 GHz) and a two-GPU-enabled Nvidia GeForce RTX Titan graphics card (24 GB of memory).

### Histological Microenvironmental Feature Extraction

Morphological image processing (MIP) involves a collection of non-linear operations related to the shape or morphology of features in an image (23). The images may contain numerous imperfections. In particular, the binary regions produced by simple thresholding are distorted by noise and texture. To identify the features of interest in whole-slide images after removing inevitable imperfections, feature extraction of the histological microenvironment was performed based on MIP, which is generated by optimizing the structuring element (SE) over the image in an activity similar to convolution (23). At each pixel position, an individual operation was applied between the corresponding SEs and the matrix data of each pathology image. The successive operations of morphological erosion and dilation of MIP were performed based on the nature of the SE (24). The PTS area was calculated as the sum of pixels of stroma tissue within the tumor region boundaries derived from MIP. Then, it was adjusted by the tumor area computed using the total number of annotated pixels originating from cancer. The PTS score is defined as [PTS area]/[tumor area]. An overview schematic of the analysis is displayed in **Supplementary Figure 1**.

### Statistical Analysis

All analyses were performed with R statistical software (version 4.0.0) and Python (version 3.6.9). Demographic differences between the two groups were tested using the Student’s t-test and Pearson chi‐square test. To assess the performance of the proposed parameter, we obtained the area under the curve of the receiver operating characteristic (ROC), a distribution of the performance metric. We used an unadjusted logistic regression method and calculated odds ratios (ORs) and their 95% confidence intervals (CIs) to assess statistical associations between independent variables and outcomes. Two‐sided *P ≤ 0.05* was considered to be statistically significant.

## Results

### Image Processing

**Figure 1:**
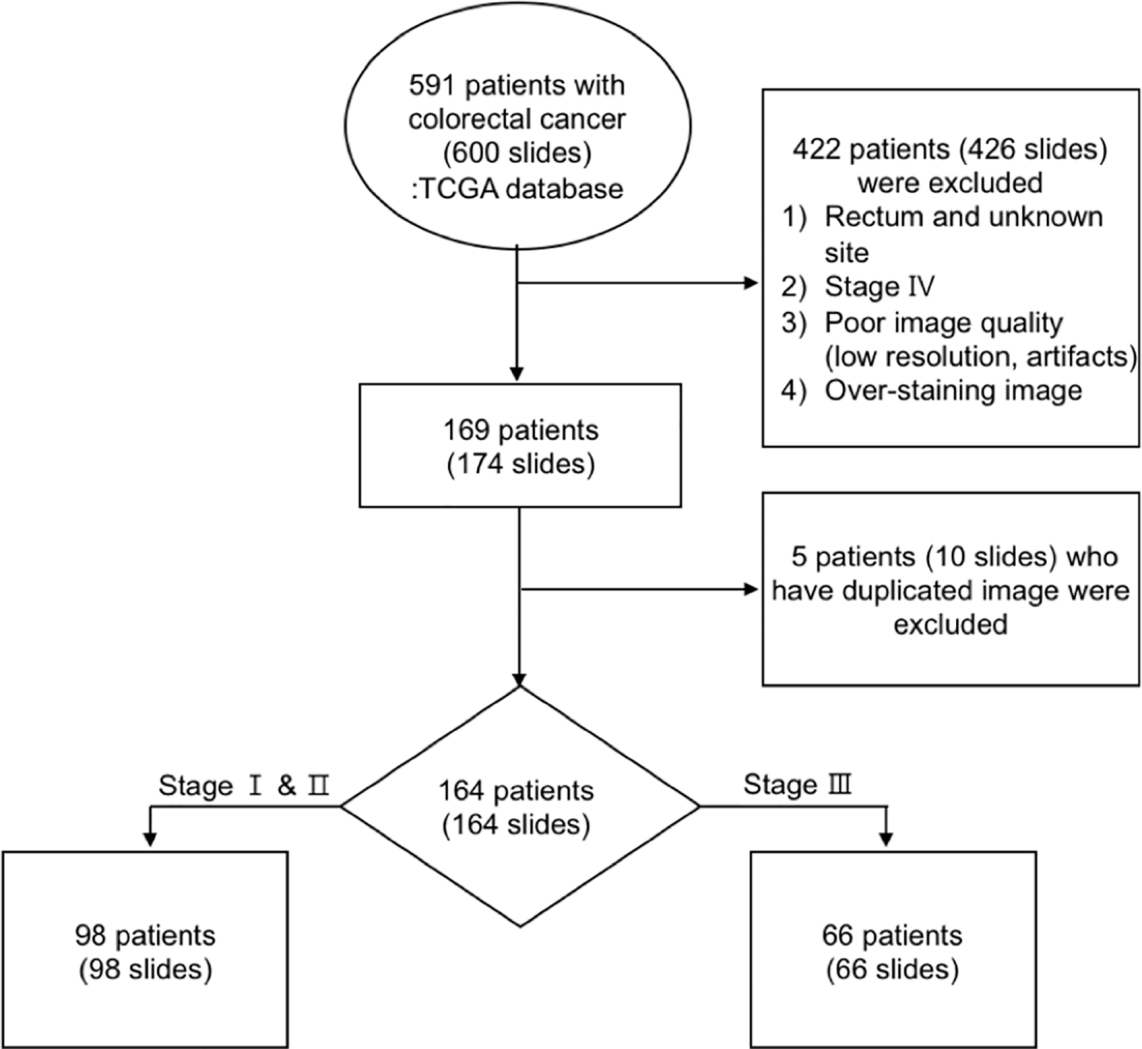
Workflow of image data processing.

A total of 591 patients (600 slides) with CRC in TCGA data were collected, of which about 153 patients (154 slides) were excluded from the study because they were diagnosed with rectal cancer. After excluding 210 patients (217 slides) with inadequate image data, such as poor image quality, bad H&E staining (i.e., too weak or too strong), duplicated images, and artifacts, and 64 patients (65 slides) with distant metastases, 164 patients (164 slides), with a diagnosis of colon cancer in stages I, II, and III based on the seventh edition of the AJCC, were analyzed (25). The image data processing workflow is shown in **Figure 1**.

### Patient Characteristics

A total of 98 patients (59.8%) were in the LNM-negative group, while the LNM-positive group accounted for 40.2% (**Table 1**). The patients without LNM {mean age 66.8 years [standard deviation (SD), 13.1]} were older than those with LNM [mean age 61.8 years (SD, 13.6), *P* = 0.021] (**Table 1**). Among the patients with LNM, 54.5% were female, and 45.5% were male (**Table 1**). A total of 66 patients with LNM had T3 (80.3%) and T4 (15.2%) primary tumors, while T1 and T2 (4.5%) tumors were less frequent (*P* < 0.001). According to the data, LI and VI were more likely to occur in the patients with LNM, and 42 cases (63.6%) and 25 cases (37.9%), respectively, were observed in our sample (all *P* < 0.001, **Table 1**). The details of the patients’ clinicopathological summaries are shown in **Table 1**.

**Table 1:** Clinical and pathologic characteristics of the included patients with colon cancer.

		LNM-negative	LNM-positive	*P*-value
		(N = 98)	(N = 66)	
Age, yr, mean (SD)		66.8 (13.1)	61.8 (13.6)	0.021
Sex, n (%)				0.777
	M	48 (49.0)	30 (45.5)	
	F	50 (51.0)	36 (54.5)	
Race, n (%)				0.075
	Asian	7 (7.1)	0 (0.0)	
	Black or African American	23 (23.5)	19 (28.8)	
	White	68 (69.4)	47 (71.2)	
T stage, n (%)				
	T0	1 (1.0)	0 (0.0)	<0.001
	T1	4 (4.1)	1 (1.5)	
	T2	25 (25.5)	2 (3.0)	
	T3	63 (64.3)	53 (80.3)	
	T4	5 (5.1)	10 (15.2)	
N stage, n (%)				<0.001
	N0	98 (100.0)	0 (0.0)	
	N1	0 (0.0)	46 (69.7)	
	N2	0 (0.0)	20 (30.3)	
LI, n (%)				<0.001
	Yes	11 (11.2)	42 (63.6)	
	No	79 (80.6)	22 (33.3)	
	NA	8 (8.2)	2 (3.0)	
PI, n (%)				
	Yes	12 (12.2)	13 (19.7)	0.208
	No	58 (59.2)	32 (48.5)	
	NA	28 (28.6)	21 (31.8)	
VI, n (%)				<0.001
	Yes	13 (13.3)	25 (37.9)	
	No	76 (77.6)	37 (56.1)	
	NA	9 (9.2)	4 (6.1)	
AnyI, n (%)				
	Yes	23 (23.5)	45 (68.2)	<0.001
	No	68 (69.4)	20 (30.3)	
	NA	7 (7.1)	1 (1.5)	

SD, standard deviation; NA, not applicable; LI, lymphatic invasion; PI, perineural invasion; VI, venous invasion; AnyI, any feature of extramural tumor deposits.

### Evaluation of Histological Image Segmentation Using CNN

**Figure 2:**
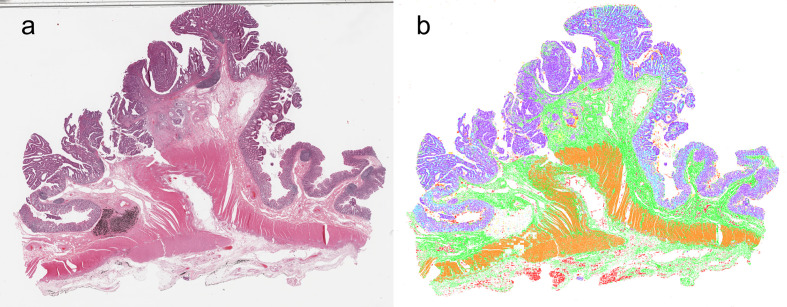
Synthetic whole-slide images. **(A)** original pathology image. **(B)** Visualization of image segmentation by the model: colon cancer epithelium, purple; stroma, green; normal epithelium, light blue; muscle, orange; adipose tissue, red; lymphocyte, blue; mucus, yellow.

In **Supplementary Figure 2**, we present the curves of training accuracy, loss, and test accuracy over epochs. The training and test accuracy curves converge on approaching 56 epochs, where training met our criterion for termination. The proposed model achieved high segmentation performance, scoring a test mean DSC of 0.892. We observed balanced class performance for all the neural networks we tested (including other architectures), with DSC values of 0.938, 0.968, 0.841, 0.732, 0.928, 0.815, and 0.930 for adipose tissue, lymphocytes, mucus, smooth muscle, normal colon mucosa, stroma, and colon cancer epithelium, respectively (**Table 2**). A representative image of the corresponding segmented classes from the designed model is shown in **Figure 2**.

**Table 2:** Predictive values of the PTS score for metastasis in colon cancer.

DSC score	mean (SD)	95% CIs	
		Lower	Upper
Adipose tissue	0.938 (0.141)	0.93	0.946
Lymphocytes	0.968 (0.055)	0.964	0.972
Mucus	0.841 (0.182)	0.83	0.852
Smooth muscle	0.732 (0.313)	0.707	0.758
Normal colon mucosa	0.928 (0.094)	0.921	0.935
Stroma	0.815 (0.235)	0.793	0.837
Colon cancer epithelium	0.930 (0.097)	0.925	0.936
Total	0.892 (0.179)	0.888	0.897

DSC, dice similarity coefficient; SD, standard deviation; CIs, confidence intervals.

### PTS Score for Prognostic Factor

**Figure 3:**
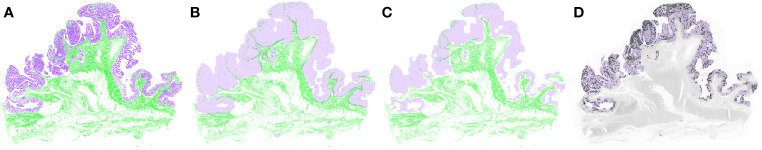
Tumor and peri-tumoral stroma (PTS) segmentation after applying morphological image processing. **(A)** The model extracted the tumor and stromal portion from the whole-slide image. After the morphological dilation **(B)** and erosion operations **(C)**, the PTS area was segmented **(D)**. Purple represents tumor, and green represents PTS.

**Figure 3** shows the PTS and tumor segmented through MIP. The mean score for the PTS was 0.380 (SD 0.285), and significantly higher PTS scores were observed in the LNM-positive group than in the LNM-negative group (*P* < 0.001) (**Table 3**). In the univariate analyses, the PTS scores for patients of the LNM-positive group were significantly higher than the scores of those of the LNM-negative group (OR 26.654; CIs 5.677–196.987, *P* < 0.001) (**Table 3**). The PTS score had a moderate ability to identify the presence of LNM in colon cancer (AUC 0.677; CIs 0.593–0.760) (**Supplementary Figure 3**).

**Table 3:** Predictive values of the PTS score for metastasis in colon cancer.

PTS score		mean (SD)	*P*-value	Univariate logistic regression			
				OR	95% CIs		*P*-value
LNM			<0.001				<0.001
	Negative	0.228 (0.160)		Ref.			
	Positive	0.380 (0.285)		29.654	5.677	196.987	
LI			0.004				0.002
	no	0.245 (0.163)		Ref.			
	yes	0.380 (0.308)		14.199	2.992	82.384	
PI			0.469				0.463
	no	0.258 (0.185)		Ref.			
	yes	0.228 (0.186)		0.369	0.019	4.283	
VI			0.274				0.159
	no	0.272 (0.186)		Ref.			
	yes	0.334 (0.328)		2.927	0.635	13.459	
AnyI			0.040				0.035
	no	0.255 (0.168)		Ref.			
	yes	0.337 (0.287)		5.050	1.200	24.847	

PTS, peri-tumoral stroma; SD, standard deviation; OR, odds ratio; CIs, confidence intervals; LNM, lymph node metastasis; Ref., reference; LI, lymphatic invasion; PI, perineural invasion; VI, venous invasion; AnyI, any feature of extramural tumor deposits.

Compared with the patients who did not have EMTDs, the PTS scores in LI and AnyI were significantly higher in the patients with colon cancer (*P* < 0.001 for both), while no significant association was identified between the PTS score and LI, VI, or AnyI (**Table 3** and **Supplementary Figure 4**). Furthermore, we performed subgroup analysis to evaluate the effect of the stage of tumor penetration (T-stage) on the predictive value of the PTS scores. Comparing the PTS scores for patients in the T3–4 stages, the univariate logistic regression model demonstrated that the ORs were 16.415 (CIs 2.912–124.106, *P* = 0.003) for LI, 0.549 (CIs 0.029–6.678, *P* = 0.659) for PI, 3.106 (CIs 0.643–15.753, *P* = 0.156) for VI, and 5.529 (CIs 1.123–34.790, *P* = 0.049) for AnyI; in contrast, for patients in the LNM-negative group, no significant difference was observed in terms of the prediction ability of the PTS scores in colon cancer with early T-stages on LI, VI, PI, or AnyI (**Table 4**).

**Table 4:** Predictive values of the PTS score for metastasis stratified by T stage in colon cancer.

PTS score		T0, T1, T2						T3, T4					
					
		Univariate logistic regression						Univariate logistic regression					
		mean (SD)	p-value	OR	95% CIs		*P*-value	mean (SD)	p-value	OR	95% CIs		*P*-value
LI			0.524				0.511		0.005				0.003
	no	0.244 (0.191)		Ref.				0.246 (0.153)		Ref.			
	yes	0.300 (0.182)		4.568	0.035	445.833		0.391 (0.320)		16.415	2.912	124.106	
PI			NA				NA		0.661				0.659
	no	0.267 (0.180)		Ref.				0.255 (0.189)		Ref.			
	yes	0.045 (NA)		NA	NA	NA		0.235 (0.186)		0.549	0.029	6.678	
VI			0.143				0.271		0.248				0.156
	no	0.246 (0.175)		Ref.				0.281 (0.190)		Ref.			
	yes	0.133 (0.090)		0.001	0.000	12.905		0.351 (0.336)		3.106	0.642	15.753	
AnyI			0.903				0.895		0.041				0.049
	no	0.253 (0.186)		Ref.				0.256 (0.161)		Ref.			
	yes	0.263 (0.192)		1.356	0.009	111.014		0.345 (0.296)		5.529	1.123	34.79	

PTS, peri-tumoral stroma; SD, standard deviation; OR, odds ratio; CIs, confidence intervals; Ref., reference; LI, lymphatic invasion; PI, perineural invasion; VI, venous invasion; AnyI, any feature of extramural tumor deposits.

## Discussion

In this study, we established a novel stromal microenvironment parameter—PTS score—to predict LNM in patients with colon cancer. To the best of our knowledge, this is the first study to develop a predictive histopathological parameter for LNM in patients with colon cancer by using artificial intelligence.

Considering the high interobserver variability in traditional pathological assessment (26–29), more accurate and reproducible histopathological assessments can reduce the inaccuracies associated with relying on subjective individual markers and better define the optimal treatment strategy for colon cancer. Whole-slide images (WSIs) contain a vast amount of information regarding cancer patients; however, it can be difficult to assess all features through manual evaluation of histology tissues, because it is significantly time consuming and can lead to substantial intra- and inter-observation variations among pathologists (30–32).

To date, a few parameters for assessing the prognosis of patients with colon cancer have been developed using computer-aided CNN methods from pathologic images (18, 33–35). In a recent German study, Kather et al. developed a parameter, the so-called “deep stroma score,” to predict the prognosis of overall survival directly from histopathological images in CRC patients (18). However, it comprised information from not only stroma (cancer-related or not cancer-related stroma) but also various other components such as debris and adipose tissue, regardless of their distance from the tumor (18). The tumor microenvironment is a heterogeneous population of cells composed of tumor cells and tumor-associated stroma, which promote tumor growth, development, and propagation, surrounding non-cancer or stromal cells recruited by the tumor (36–39). Therefore, an analysis focused on the stromal components near a tumor could appropriately assess the contribution of cancer-related stroma in cancer tissue.

Another study by Takamatsu et al. presented a deep-learning model for predicting LNM from pathology images with cytokeratin immunohistochemistry in early CRC (33). However, only a limited number of LNM events exist, which limited the data available for the study, caused by the low rate of metastasis in early CRC. Furthermore, stepwise procedures, such as cytokeratin immunohistochemical staining and calculation of several parameters followed by model selection, were necessary for the prediction. Thus, it is inconvenient and not applicable for unusual cases that lack typical histological features in a slide image. A recent Chinese study also presented several morphologic parameters from pathology data to predict recurrent risk in stage III CRC (34). The authors generated new parameters by combining different histological components from whole tissue slides. Although this might be an interesting attempt, the validity of the parameters is not guaranteed considering the variability of tissue components contained in histological images through the pathology preparation. Lastly, Bychkov et al. stratified CRC patients for disease-specific survival into low- and high-risk groups using a CNN method on pathology images (35). However, they did not present any specific histological prognostic parameter, which could have potential utility in clinical decision-making.

Several previous studies have revealed prognostic information regarding the tumor-stroma ratio in CRC (40–46). Despite the evidence, it has not been implemented in routine pathology reporting because of significant variations in methodology and the lack of a standardized procedure for assessing tumor-stroma ratios. Published studies propose manual assessments of the deepest point of tumor invasion (40–42, 45, 46), systematic random point assessment (43), and the use of a semi-automatic method combining human input and a deep-learning algorithm using WSIs (44). However, time- and labor-intensive manual evaluations by pathologists with expertise must take precedence in these methods.

To overcome the issues discussed above, herein we applied a scoring procedure in which the relative amounts of tumor and the PTS score, as a straightforward measure, were calculated and adjusted based on the entire tumor area in a WSI using a CNN. Even without deep domain knowledge and the experience to assess pathologic images, this allows for obtaining easy and reproducible quantification of PTS and has the potential to pave the way for the implementation of the PTS score in clinical practice.

Our findings also indicated that the PTS score may be an independent parameter for predicting the presence of EMTDs in colon cancer. Histopathological identification of LI, VI, and PI in cancer tissue has long been recognized as a potential prognostic indicator for patient outcomes because of the likely association with progression to lymphatic metastasis (29, 47, 48). It is necessary to identify the parameters that can reduce interobserver variability because the rate of LI, VI, and PI detection is directly related to both technical aspects of tissue preparation, such as staining technique and the number of blocks examined, and the pathologist’s experience and specialization (49, 50).

The present study demonstrated a significant association between the PTS score and LI and AnyI, whereas no prognostic significance for VI and PI were found. Taken together, the PTS score could be a useful tool to identify patients who are at risk of developing LNM and EMTDs in colon cancer. Despite these advantages, the current study has a few limitations. One limitation of this study is that cases submitted for the TCGA database might be biased in terms of mainly including images in which the morphological patterns of disease are definitive, which could be different from what pathologists encounter in their daily practice. In addition, we could not alleviate the heterogeneity in stain color, despite the well-established stain normalization method. Therefore, it will be necessary to standardize a pathology stain method, which will decrease the difficulty of producing consistent diagnostic results and help build systems that generalize well. Another limitation is that, despite a good potential prognostic value of the PTS score for LNM and EMTDs overall and in T3–4 colon cancer patients, a prognostic value for cases with early T-stages was not observed. This may result from a class imbalance problem due to the limited sample size and the low event rate of EMTDs. Therefore, further studies should be performed using larger samples to obtain more accurate results for early T-stage colon cancer.

In conclusion, we established that the PTS score is, potentially, a promising and easy-to-apply prognostic parameter for LNM in colon cancer. However, because of the limitations inherent in studies based on observational data, these findings should be confirmed through subsequent prospective clinical trials.

## Data Availability Statement

The datasets presented in this study can be found in online repositories. The names of the repository/repositories and accession number(s) can be found in the article/**Supplementary Material**.

## Ethics Statement

This study was reviewed and approved by the Institutional Review Board of the Kyung Hee University Hospital at Gangdong (KHNMC IRB 2020-09-025). The need for informed consent was waived because all data used in this study were de-identified.

## Author Contributions

MK designed the study. JY and MK analyzed and interpreted the data and wrote the manuscript. JC, HK, JJ, and JY supervised the project and revised the paper. All authors contributed to the article and approved the submitted version.

## Funding

This research was supported by the Basic Science Research Program of National Research Foundation of Korea (NRF), which is funded by the Korean Ministry of Science, ICT and Future Planning (grant number: NRF-2019R1C1C1003524).

## Conflict of Interest

The authors declare that the research was conducted in the absence of any commercial or financial relationships that could be construed as a potential conflict of interest.

## Supplementary Material

Supplementary Figure 1 Overview of the analysis pipeline.

Supplementary Figure 2 Performance plot of the proposed convolutional neural network model showing training accuracy (green line), test accuracy (yellow line), training loss (red line), and test loss (blue line).

Supplementary Figure 3 Estimated binormal ROC curve (bold) and the 95% confidence bands (stepped red line) of the ROC curve for assessing the predictive value of the peri-tumoral stroma score.

Supplementary Figure 4 Differences of the peri-tumoral stroma score according to the status of extramural tumor deposits in colon cancer (**P* ≤ 0.05; ***P* ≤ 0.01).
