# Supplementary figures and images for: Deep Convolutional Neural Network-Based Lymph Node Metastasis Prediction for Colon Cancer Using Histopathological Images
Source: Front Oncol. 2021 Jan 13;10:619803. doi: 10.3389/fonc.2020.619803 (PMC7838556; doi:10.3389/fonc.2020.619803)

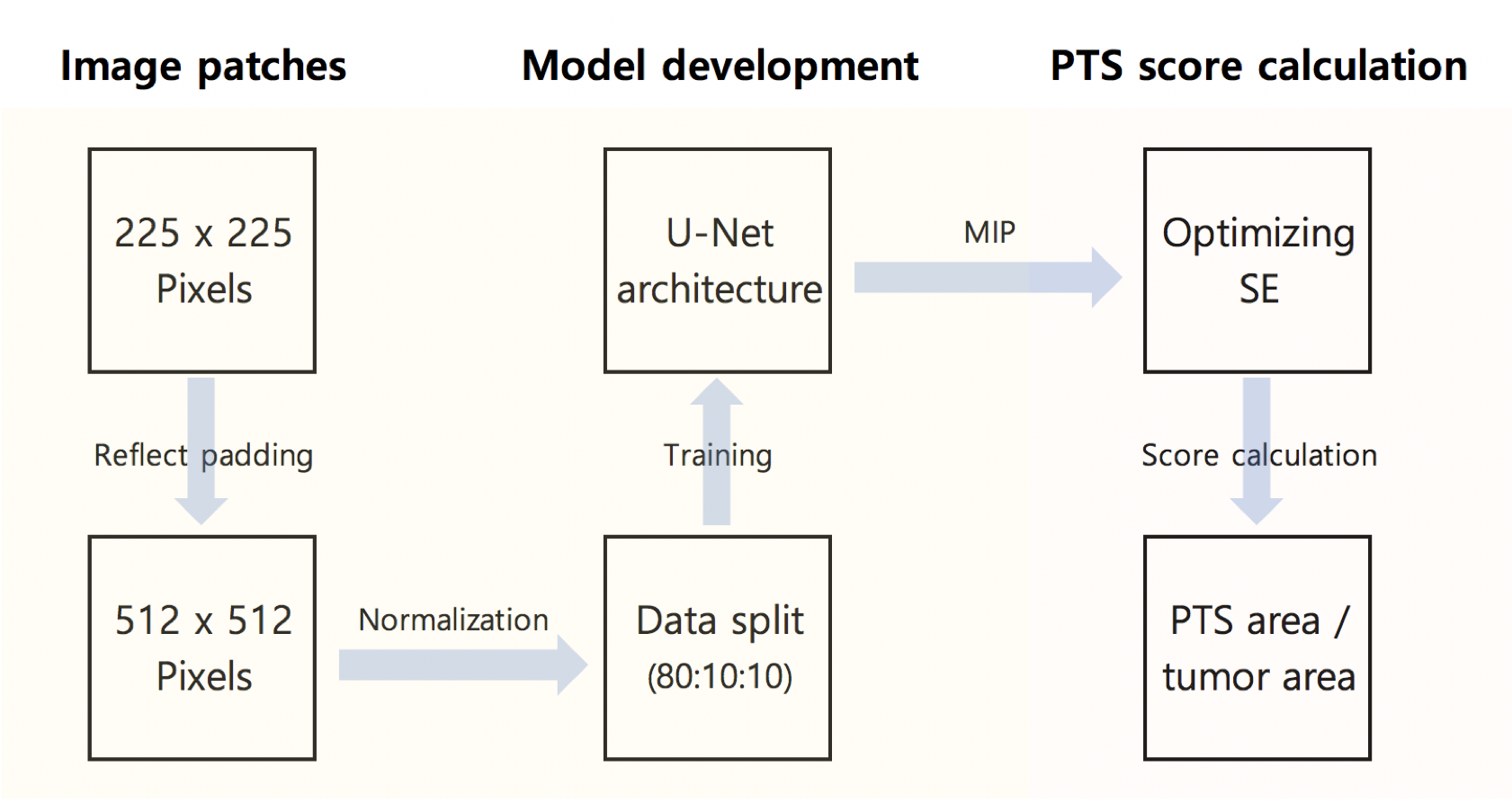

Supplement: Supplementary Figure 1 — Overview of the analysis pipeline. [file Image_1.png]

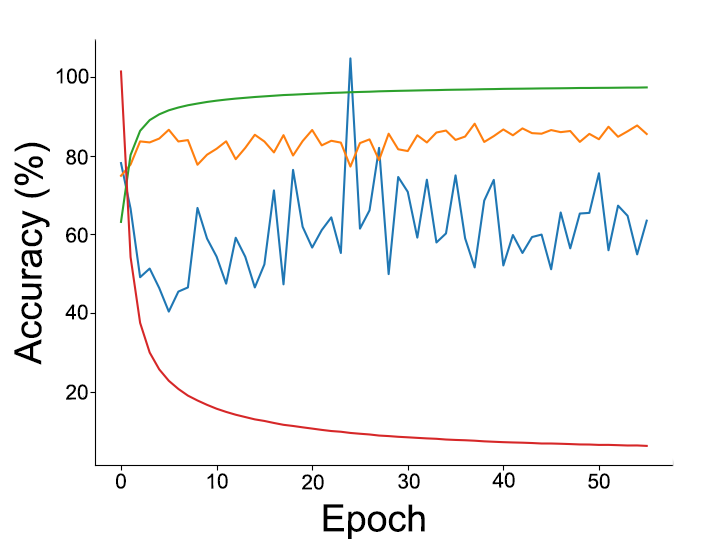

Supplement: Supplementary Figure 2 — Performance plot of the proposed convolutional neural network model showing training accuracy (green line), test accuracy (yellow line), training loss (red line), and test loss (blue line). [file Image_2.png]

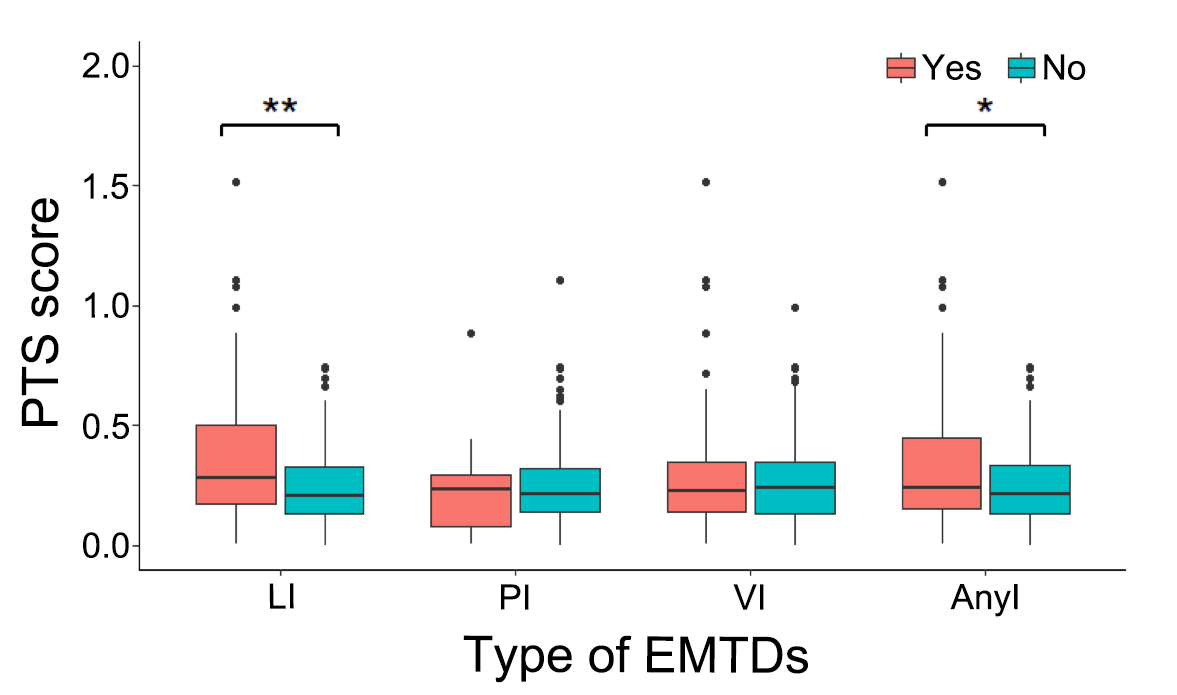

Supplement: Supplementary Figure 3 — Estimated binormal ROC curve (bold) and the 95% confidence bands (stepped red line) of the ROC curve for assessing the predictive value of the peri-tumoral stroma score. [file Image_3.png]

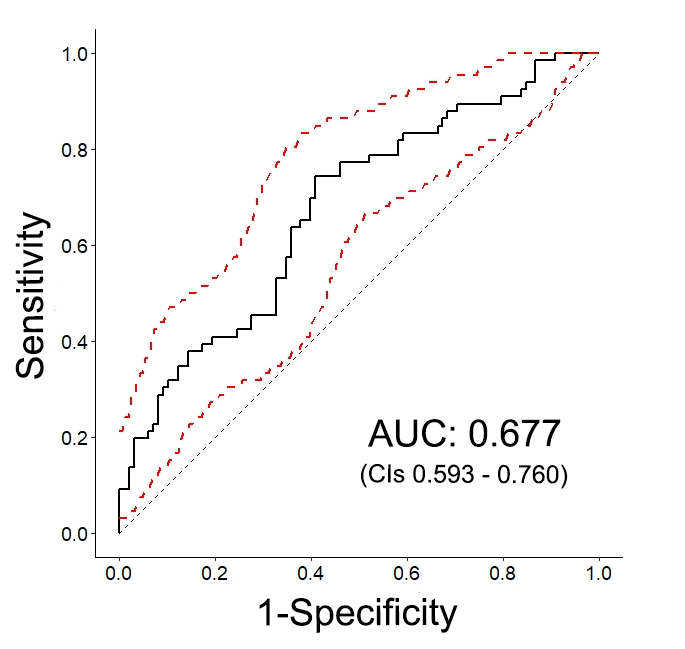

Supplement: Supplementary Figure 4 — Differences of the peri-tumoral stroma score according to the status of extramural tumor deposits in colon cancer (*P ≤ 0.05; **P ≤ 0.01). [file Image_4.png]
